# Uncharted Territories: Dynamic Hip Screw Migration Into the Pelvis Requiring Laparotomy

**DOI:** 10.7759/cureus.62810

**Published:** 2024-06-21

**Authors:** Daniel A Lewandowski, Martin Clarkson, Aso Mohammed

**Affiliations:** 1 Trauma and Orthopaedics, University Hospital of Wales, Cardiff, GBR; 2 Trauma and Orthopaedics, Morriston Hospital, Swansea, GBR

**Keywords:** vascular surgery laparotomy, general surgery laparotomy, trauma laparotomy, dynamic hip screw (dhs), dynamic hip-screw fixation, dhs case report, rare dhs complication, dhs migration into pelvis, laparotomy for dhs retrieval, dynamic hip screw fixation

## Abstract

Hip fractures are common in patients with poor bone quality and are seen to affect the elderly and frail population. We report a case of implant failure after fixing an unstable intertrochanteric fracture with a dynamic hip screw (DHS). The patient presented with a DHS that had migrated into the pelvis approximately six months after surgery. Plain radiographs showed migration of the DHS through the acetabulum and into the pelvis. Migration of DHS into the pelvis is an extremely rare complication and has only been reported a few times. A 71-year-old man presented with a fall and confusion. The patient reported having a fall but could not recall the exact events. Past medical history included Alzheimer's dementia, osteoporosis, left total hip replacement, right DHS, peripheral neuropathy, and recurrent falls. He had undergone reduction and fixation of a right intertrochanteric fracture with DHS implant via direct lateral approach six months before hospital admission. On examination, he had right-sided hip pain and was unable to straighten leg raise. His abdomen was soft and non-tender, with no distension or palpable masses. Neurovascular status was normal, and no signs of infection were detected. On the anteroposterior radiograph, the implant seemed to have migrated through the acetabulum and into the abdomen. A CT of the abdomen and pelvis was performed to identify any visceral injuries (negative) and for surgical planning. The patient underwent a midline laparotomy to remove the implant. Although the exact reason for the implant failure is unknown, the migration of an unbroken hip screw into the abdomen and pelvis requiring laparotomy has not been reported in literature.

## Introduction

Because of the aging population, hip fractures are becoming more prevalent, and, within one year of operation, have shown to have high mortality rates [1,2]. Poor bone quality and fragility are significant factors for hip fractures, and those patients with osteoporosis are more frequently affected [3]. Failure of dynamic hip screws (DHS) has been previously reported in the literature, and very few cases have been reported of screw migration into the pelvis. DHS retrieval by laparotomy, to the best of our knowledge, has never been reported in the literature.

## Case presentation

**Figure 1:**
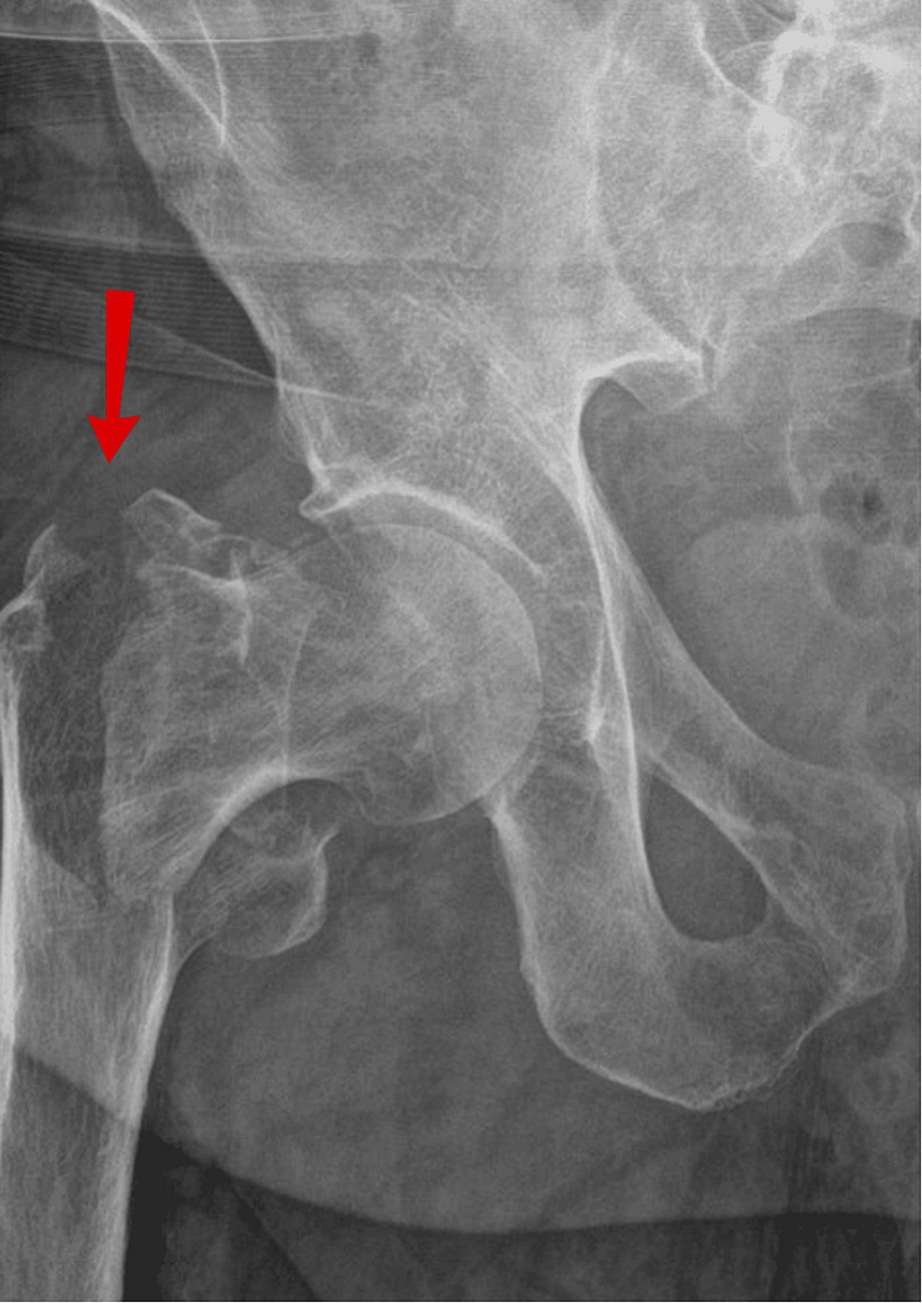
AP view X-ray of initial fracture presentation

**Figure 2:**
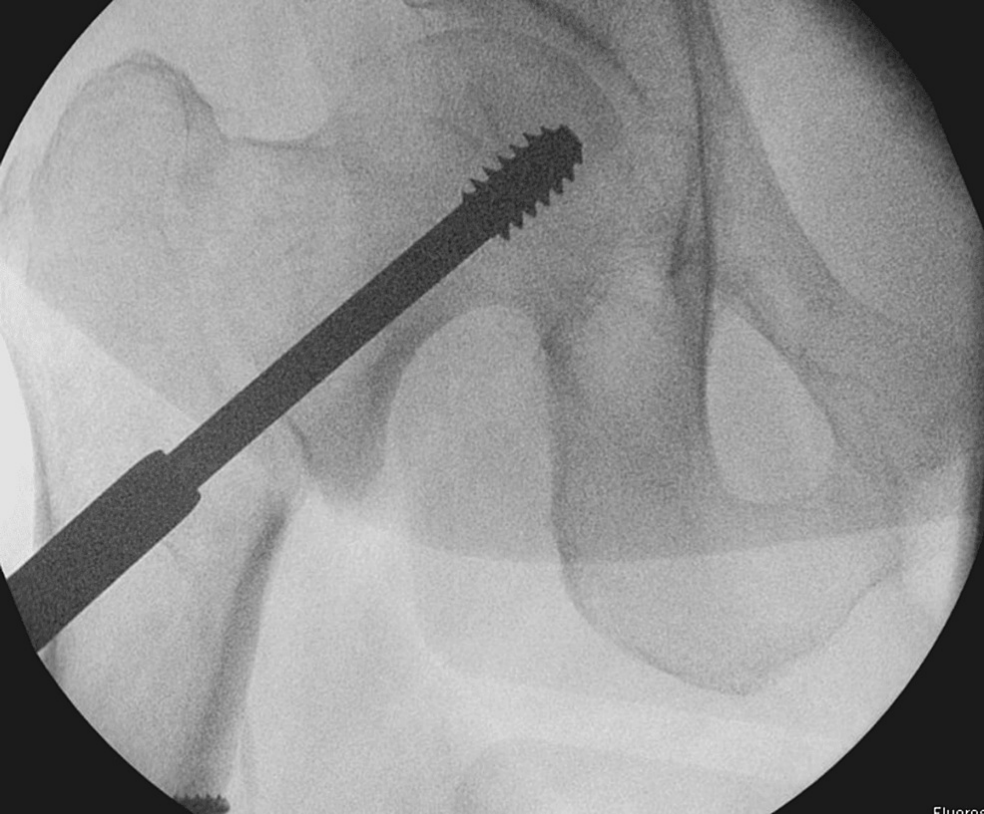
Intraoperative images of fracture fixation with a dynamic hip screw (DHS) (AP view)

**Figure 3:**
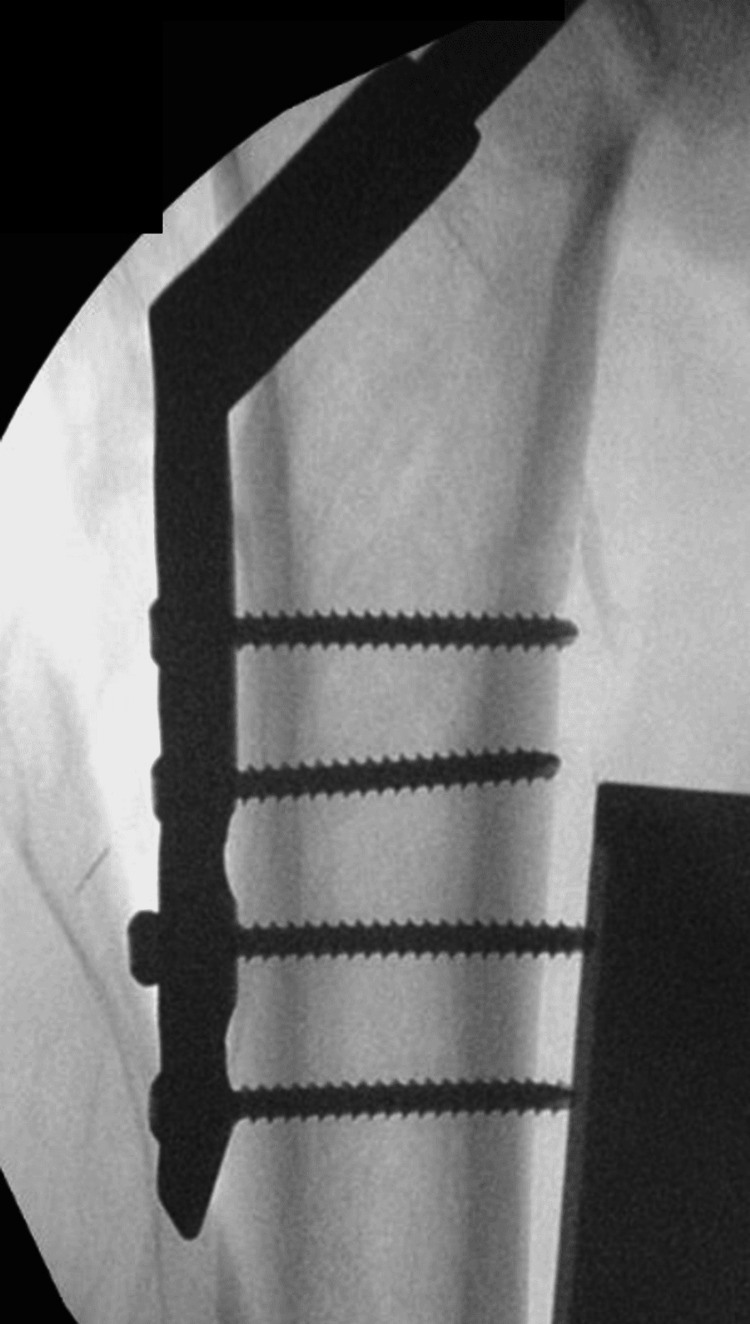
Intraoperative images of fracture fixation with a dynamic hip screw (DHS) (AP view)

**Figure 4:**
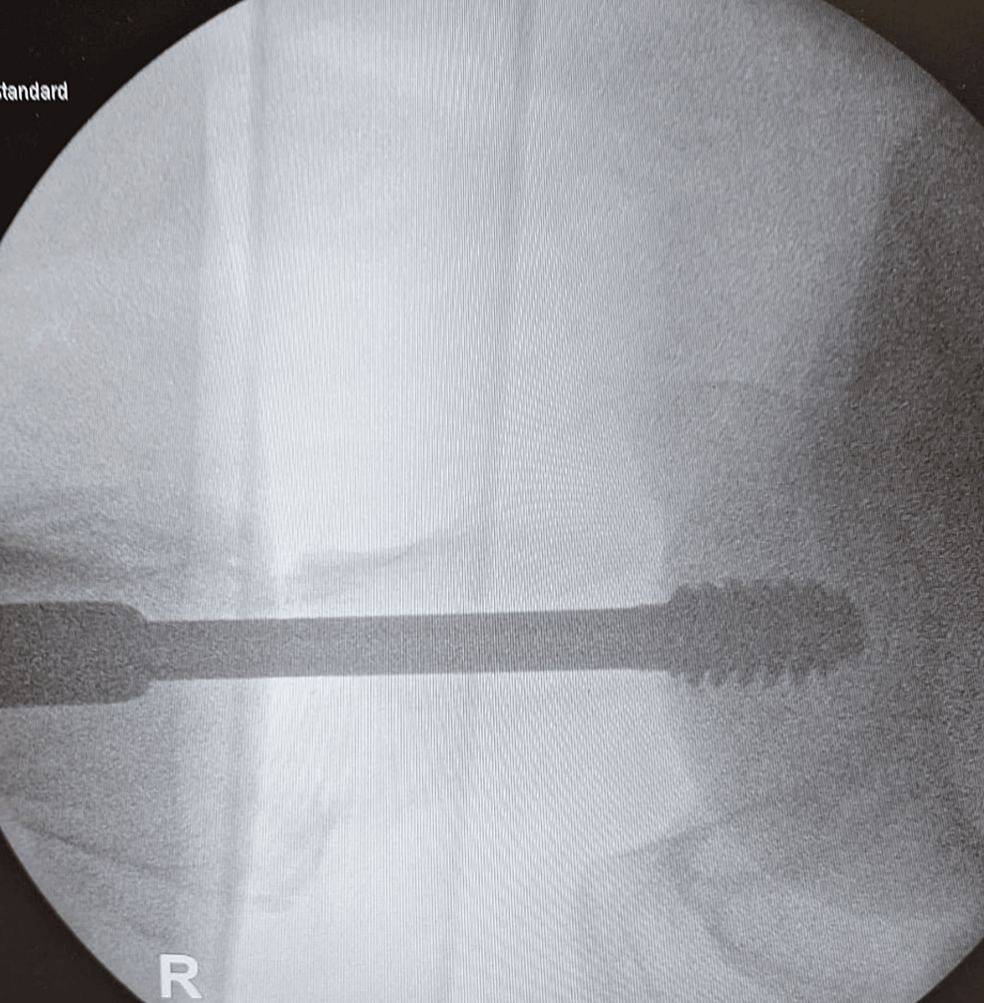
Intraoperative images of fracture fixation with a dynamic hip screw (DHS) (lateral view)

A 71-year-old man presented with a fall and confusion. The patient had reported having a fall but could not recall the events of the fall or when it happened. The ambulance crew had reported that residential home staff notified that the patient had been bedridden for the last two days. He had recently been suffering from urinary retention, and his urine had been dark and foul-smelling. The patient began developing hallucinations and episodes of confusion. The patient had a past medical history of Alzheimer's dementia, osteoporosis, bilateral knee replacements, left total hip replacement, peripheral neuropathy, and recurrent falls. He had undergone reduction and fixation of a right intertrochanteric fracture (Figure 1) classified as a type A1.2 fracture according to AO classification [4]. The fracture was fixed with a DHS implant via a direct lateral approach six months before hospital admission (Figures 2-4) and used a Zimmer frame to mobilise in respite care. On examination, the findings of his right lower limb examination were abnormal as he was unable to perform a straight leg raise. His abdomen was soft and non-tender, with no distension or palpable masses. The patient was neurovascularly intact and haemodynamically stable. No evidence of infection on examination was detected.

**Figure 5:**
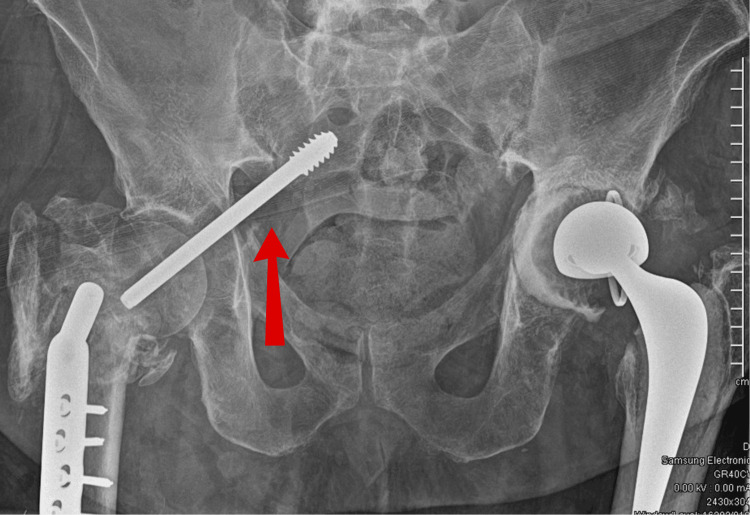
AP view X-ray showing migration of the dynamic hip screw (DHS) into the pelvis

On the anteroposterior radiograph, the implant seemed to have migrated through the acetabulum and into the pelvis/abdomen (Figure 5). A CT of the abdomen and pelvis was performed to identify any visceral injuries (negative) and for surgical planning. A further CT angiogram was conducted and showed no evidence of a pelvic haematoma or active extravasation. The angiogram was also performed to ensure that the metalwork was not near any viscera or neurovascular bundles.

After consultation with the general surgical and vascular teams, a midline laparotomy was performed to remove the implant. The screw remained covered by the peritoneum, and no visceral injury was identified. Despite antibiotic care following the operation, the patient developed intra-abdominal sepsis. This was seen as a postoperative complication, possibly because of wound infection, and the patient died in the hospital on day six following admission.

## Discussion

Intertrochanteric fractures have historically been categorised by the Evans classification, which are fractures that occur between the lesser and greater trochanters of the hip [5]. This classification was introduced in 1949 by Dr. Alfred G. Evans and helps guide treatment decisions and assess fracture stability to predict outcomes. The Evans classification divides intertrochanteric fractures into stable (type 1) and unstable fractures (type 2). Some key features of type 1 fractures include minimal displacement of the fracture, bone fragments being well aligned and stable, and the medial cortex remaining intact. Usually, these fractures can be treated with methods such as internal fixation. Type 2 fractures tend to show significant displacement, are often comminuted fractures or reverse oblique fractures, and the medial cortex is disrupted, leading to instability [5]. For these fractures, treatment is usually done with intramedullary nailing or other fixation techniques.

Since its establishment, the Evans classification has expanded to include other subtypes to further group fracture patterns and their implications. For example, type 2 fractures can be further grouped based on the degree of comminution and specific fracture patterns. This helps surgeons select the most appropriate treatment option to help with patient outcomes and reduce complication rates.

Furthermore, with intertrochanteric fractures, a measurement called tip apex distance (TAD) is crucial in orthopaedic surgery. It is important in fixations with a DHS or intramedullary nail. TAD is defined as the sum of distances from the tip of the screw to the apex of the femoral head in both anteroposterior and lateral radiographs [6]. This is an important measure for many reasons: a smaller TAD is associated with lower risks of screw cut-out, which is where the screw migrates out of the bone; a TAD less than 25 mm significantly reduces the risk of mechanical failure; TAD offers a standardised way to evaluate surgical fixations across numerous studies and cases, facilitating optimising techniques in surgery and patient care [6].

Our patient had features of a displaced type 1 fracture and was deemed suitable for internal fixation with a DHS. Intraoperative images were obtained, but because of their magnification, it was difficult to measure the true TAD in this case.

Several types of DHS failure have been reported in the literature, including hip screw breakage [7], bending of the hip screw at the screw-barrel interface without breakage [8], and breakage in the barrel of the plate and bending in the hip screw [9]. Recent studies show DHS failure rates of approximately 6.8% [10]. As noted by Spivak et al. [11], a DHS can fail in two ways. The first way is by the low-stress fatigue failure of the device, related to the design of the screw, including the length of the barrel and the internal threaded region. The second type of implant failure is by high-stress loading, usually observed in the nonunion of the intertrochanteric area. Migration of an unbroken hip screw into the abdomen and pelvis is very rare and has only been reported a few times in literature. To the best of our knowledge, the retrieval of a DHS requiring laparotomy has not been reported in the literature.

## Conclusions

Since the development of the DHS, migration into the pelvis has been a very rare complication and has only been reported a few times in literature. Furthermore, retrieval of DHS using a laparotomy has never been recorded. The recognition of such events and compliance with surgical techniques, especially when operating on elderly, osteoporotic patients, can help avoid such complications.
